# New insights into the functions of Cox-2 in skin and esophageal malignancies

**DOI:** 10.1038/s12276-020-0412-2

**Published:** 2020-04-01

**Authors:** Hyeongsun Moon, Andrew C. White, Alexander D. Borowsky

**Affiliations:** 1 Center for Immunology and Infectious Diseases, University of California, Davis, CA 95616 USA; 2 Department of Biological Sciences, Cornell University, Ithaca, NY 14850 USA

**Keywords:** Cancer models, Mechanisms of disease

## Abstract

Understanding the cellular and molecular mechanisms of tumor initiation and progression for each cancer type is central to making improvements in both prevention and therapy. Identifying the cancer cells of origin and the necessary and sufficient mechanisms of transformation and progression provide opportunities for improved specific clinical interventions. In the last few decades, advanced genetic manipulation techniques have facilitated rapid progress in defining the etiologies of cancers and their cells of origin. Recent studies driven by various groups have provided experimental evidence indicating the cellular origins for each type of skin and esophageal cancer and have identified underlying mechanisms that stem/progenitor cells use to initiate tumor development. Specifically, cyclooxygenase-2 (Cox-2) is associated with tumor initiation and progression in many cancer types. Recent studies provide data demonstrating the roles of Cox-2 in skin and esophageal malignancies, especially in squamous cell carcinomas (SCCs) occurring in both sites. Here, we review experimental evidence aiming to define the origins of skin and esophageal cancers and discuss how Cox-2 contributes to tumorigenesis and differentiation.

## Cancer: Inflammatory enzyme linked to skin and esophageal tumors

Drugs that block a pro-inflammatory enzyme implicated in cancer initiation and progression could help suppress the development of skin and esophageal cancers that arise from abnormal squamous cells (skin cells and cells lining the respiratory and digestive tracts). In a review article, Hyeongsun Moon from the University of California, Davis, USA, and colleagues discuss experimental evidence from genetically engineered mouse models demonstrating that expression of an enzyme called cyclooxygenase-2 (Cox-2) is critical to the transformation of stem and progenitor cells in the skin and esophagus into cancer cells. Cox-2 is already the target of many drugs approved for treating inflammatory conditions such as arthritis. The preclinical data suggest that the same medications, or agents directed at mediators of Cox-2 signaling, may help tamp down the inflammation that can spur tumor-prone cells into turning malignant.

## Introduction

Prevention of tumorigenesis represents an underappreciated opportunity to elicit a major impact in reducing cancer incidence and patient mortality. Moreover, inhibition of tumor initiation from cells that have already accumulated transforming mutations could provide a novel method for cancer prevention. To achieve this, it is important to fully understand and define the cells of origin for each cancer subtype and determine under which conditions a cell harboring transforming mutations is able to proliferate, invade surrounding tissues, and evade immune surveillance, leading to malignant cancer behavior.

Cyclooxygenase (Cox) enzymes are a class of molecules central to the environmental changes involved in tumor initiation^1^. In the normal, disease-free state, these enzymes contribute to cell homeostasis; however, when homeostasis is perturbed by disease, they play critical roles in response but can also contribute to the development of a myriad of diseases, including cancer^1^. They provide a critical function as components in the enzymatic conversion of arachidonic acid to one of five unique prostanoid molecules. Two functionally redundant Cox enzymes exist, but each is unique in its spatial and temporal expression. Cox-1 is expressed as a housekeeping enzyme in most tissues during homeostasis, whereas Cox-2 is generally upregulated only in pathological conditions such as inflammation and cancer. Moreover, increased synthesis of prostanoids through Cox-2 activity can significantly contribute to the induction of inflammation and tumorigenesis^1^. Importantly, Cox-1/2 activity can be pharmacologically inhibited by nonsteroidal anti-inflammatory drugs (NSAIDS), such as naproxen or indomethacin, and Cox-2 activity can also be suppressed by selective inhibitors. The FDA-approved Cox-2 inhibitor celecoxib is used clinically to treat inflammatory diseases such as rheumatoid arthritis and osteoarthritis^2^. Although many studies have suggested the potential benefit of Cox-2 inhibition, especially in the prevention of colon cancer, whether suppression of this enzyme can be preventative or therapeutic for squamous cell carcinomas (SCCs) remains unclear. Here, we review the cellular origins of major primary cutaneous and esophageal cancers and discuss experimental evidence for two new roles of Cox-2 in the genesis of cutaneous and esophageal SCCs based on defined genetically engineered mouse models.

## Cancer cells of origin and Cox-2 in cutaneous cancers

### Diversity of cellular origins for cutaneous cancer development

The skin is the largest organ and is tasked with protecting our body. It is also the most common site of cancer diagnosis. Primary malignancies in the skin include basal cell carcinoma (BCC), squamous cell carcinoma (SCC), and melanoma. Nonmelanoma skin cancers (keratinocyte carcinomas: BCCs and SCCs) are the most common, as over 3 million new cases are expected annually^3^. Melanoma accounts for less than 5% of overall skin cancer cases, but the incidence of melanoma is increasing, with twice as many cases in the US this year compared to 30 years ago^4,5^. Additionally, despite the much lower incidence of melanoma than nonmelanoma skin cancers, melanoma accounts for the majority of skin cancer mortality^5^.

During the last decade, there has been great progress in understanding the cellular origins of various types of cancers, including both nonmelanoma and melanoma skin cancers. Particularly, genetic approaches with lineage tracing methods in mouse models have determined the earliest steps of tumorigenesis from stem/progenitor cells in multiple organ sites^6–8^. Notably, genetically engineered mouse models using conditional knock-in and knockout systems, which specifically target each cellular subset (including interfollicular basal progenitors, hair follicle stem cells, transit amplifying cells and melanocyte stem cells), can allow us to monitor the earliest stages of nonmelanoma and melanoma skin cancer development^9^.

BCCs, the most common skin cancer, were postulated to originate from hair follicles due to the similarity in histologic characteristics of tumor tissues. However, oncogenic activation of the Hedgehog pathway revealed that both the interfollicular epidermis and hair follicular epithelium can form BCCs^10–13^. Constitutive activation of the G protein-coupled receptor Smoothened (SMO) through the *Rosa26-SmoM2* allele appeared to be primarily involved in tumor formation from basal stem/progenitors within the interfollicular epidermis and infundibulum^10^. On the other hand, genetic inhibition of the tumor suppressor Patched 1 (PTCH1) using *Ptch1*^*+/−*^ mice or expression of mutant GLI family zinc-finger 2 (GLI2, also known as glioma-associated oncogene family zinc-finger 2) using *Rosa26-LSL-rtTA; tetO-GLI2ΔN* mice demonstrated a significant contribution of keratin 15 (KRT15), keratin 19 (KRT19) and leucine-rich repeat-containing G protein-coupled receptor 5 (LGR5)-positive hair follicle stem cells in BCC development^11–13^. These studies reported that the constitutive activation of the Hedgehog pathway by oncogenic driver mutations (gain-of-function) or the absence of Hedgehog pathway suppressors could be involved in BCC formation from multiple cellular origins via resident stem/progenitor cells in both the hair follicular epithelium and interfollicular epidermis, especially in mechanosensory hot spots^11^.

**Fig. 1:**
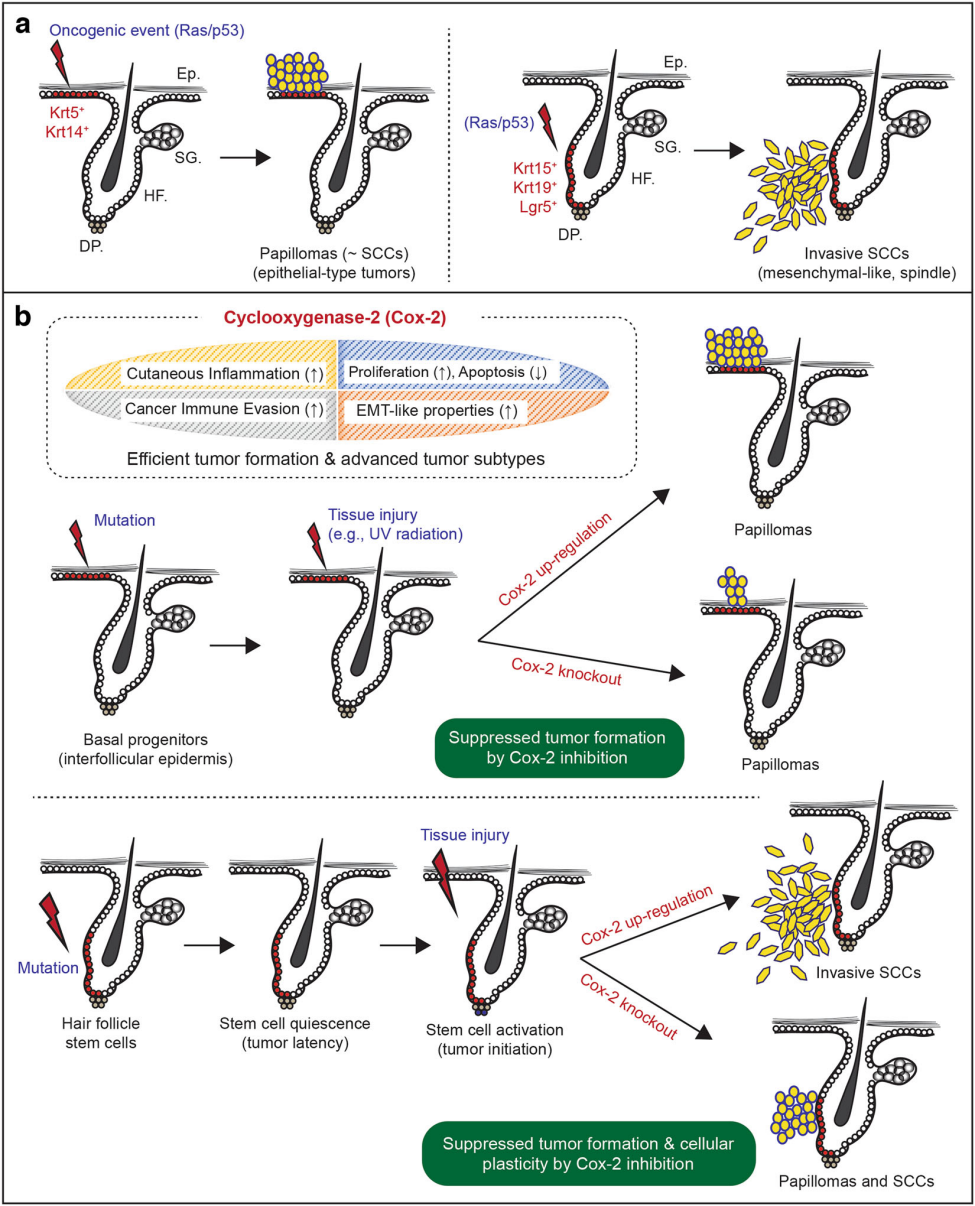
The role of Cox-2 in stem/progenitor cells during the earliest stages of cutaneous SCCs. **a** Oncogenic expression of *Ras* (gain-of-function) and *p53* (loss-of-function) can induce papillomatous tumors from basal stem/progenitors at the interfollicular epidermis. The same oncogenic combination (*Ras/p53*) in hair follicle stem cells can directly induce a more invasive form of SCC, mesenchymal-like spindle cell carcinoma. Ep., epithelium; SG., sebaceous gland; HF., hair follicle; DP., dermal papilla. **b** Skin damage, for example, UV exposure-induced skin damage, can accelerate tumorigenesis via Cox-2 upregulation from tumor-prone stem/progenitor cells. However, cell-type-specific knockout of Cox-2 can suppress tumor formation from both epidermal basal stem/progenitors and hair follicle stem cells. Furthermore, Cox-2 inhibition can suppress the cellular plasticity of hair follicle stem cell-originating cutaneous SCCs and lead to the formation of less aggressive SCC subtypes.

SCCs, unlike BCCs, have long been postulated to arise from the differentiated squamous cell layer of the interfollicular epidermis rather than hair follicles due to their histological signature, which resembles the epidermis. However, similar to BCCs, experimental murine models demonstrate that cutaneous SCCs appear to arise from both the interfollicular epidermis and hair follicles. Furthermore, interestingly, different cellular populations that are located in distinct stem cell niches throughout the epidermis and hair follicles appear to have differential tumorigenic potential when they express the same oncogenic combination. One often observed mutant signature of SCCs includes oncogenic activation of the RAS GTPase (RAS)^14–16^. Tumorigenesis associated with the cutaneous application of 7,12-dimethylbenzanthracene and 12-O-tetradecanoylphorbol-13-acetate (DMBA/TPA), the most common chemical treatment used to induce SCC in a murine model system, is primarily caused by mutations in *Hras*^15^*. Kras* mutations are also induced by this chemical mutagen but at a significantly lower frequency^15^. In addition to DMBA-induced chemical mutations, various studies have documented tumorigenesis of SCC via genetic enhancement of the RAS pathway using the *LSL-Kras-G12D* allele (constitutively activated form of *Kras*; gain of function) together with several different inducible Cre transgenic mouse models, including *Krt5-tet-on*, *Krt14-CrePR*, *Krt14-CreER*, *Krt15-CrePR*, *Lgr5-CreER* and *Lgr6-CreER*^17–23^. Oncogenic RAS expression through both DMBA chemical treatment and *Kras* gain-of-function can lead to the development of papillomatous tumors, which are considered a potential precursor lesion of SCCs. In addition, the expression of *Kras*^*G12D*^ together with loss of function of the tumor suppressor *p53* (oncogenic *Ras/p53* combination) significantly accelerates tumor transformation from benign papillomatous tumors to invasive, spindle cell SCCs^20,21^. Intriguingly, upon oncogenic *Ras/p53* expression, while *Krt5-CreER* and *Krt14-CreER* basal progenitors at the interfollicular epidermis primarily develop into papillomatous tumors, *Lgr5-CreER*, *Krt15-CrePR*, and *Krt19-CreER* hair follicle stem cells develop into invasive, mesenchymal-type SCCs^20–23^. Compared with *Lgr5-* and *Krt15*-positive hair follicle stem cells, *Lgr6-CreER* hair follicle stem cells located at the upper portion of hair follicles are less tumorigenic upon the same oncogenic *Ras/p53* expression^16,23^. Hence, these studies suggest that multiple stem cells that differentiate into hair follicular epithelium and epidermal keratinocytes can contribute to SCC formation; however, each stem cell population located in different stem cell niches may have different tumorigenic potential and contribute to the diversity of SCC subtypes even when they harbor the same oncogenic combination (summary diagrams in Fig. 1a).

While BCC and SCC originate from skin keratinocytes, cutaneous melanoma arises from melanocytes, the pigment-producing cells. Benign nevi may be precursor lesions that can progress to malignant melanocytic tumors when they gain additional mutations or genetic alterations^24,25^. Constitutive activation of the mitogen-activated protein kinase (MAPK) and extracellular signal-regulated kinase (ERK) pathways by oncogenic mutations in RAS and rapidly accelerated fibrosarcoma (RAF) genes (e.g., mutant *Braf*^*V600E*^ expression) often causes oncogenic senescence in melanocytes. These benign nevi are known to require additional genetic changes, such as the loss of tumor suppressors, including cyclin-dependent kinase inhibitor 2A (CDKN2A) and phosphatase and tensin homolog (PTEN)^24,25^. The additional genetic alterations help benign melanocytic nevi cells overcome oncogenic senescence to become malignant melanocytic tumor cells. Cutaneous melanomas, however, are often diagnosed from patients who have no clinical history of benign moles or an identifiable precursor lesion^26,27^. These melanoma cells originating from clear skin are considered to originate from sustained unrecognized benign nevi or tumor-prone melanocyte stem cells. Recent studies driven by independent groups have experimentally demonstrated that melanoma can directly originate from melanocyte stem cells using *Tyr-CreER* and *c-Kit-CreER* promoters^28–30^. These independent studies^28–30^ used the same cell-type-specific expression of oncogenic profiles: a tumor driver mutation, BRAF (*Braf*^*V600E*^ conditional knock-in allele), together with absence of the tumor suppressor PTEN (*Pten* conditional knockout allele). This combination is known to be able to induce invasive and metastatic melanoma cells^31^. Interestingly, melanoma cells directly originated from melanocyte stem cells in the absence of benign nevi. Furthermore, these melanoma-competent/susceptible melanocyte stem cells located within the hair follicles were able to translocate to the interfollicular epidermis, where they, in turn, formed cutaneous melanoma throughout the epidermis^28–30^. These new observations suggest the need to determine specific prevention strategies for melanomas arising from benign nevi and tumor-prone stem cell populations.

### The role of Cox-2 in stem cell-originating cutaneous tumor formation

Multiple resident stem cell populations within the skin layers can be involved in nonmelanoma and melanoma skin cancer development; however, each population located in distinct stem cell niches has different tumorigenic potential that can correlate with distinct tumor phenotypes. These distinctive tumorigenic capacities may be due to various reasons, including the differential intrinsic expression levels of certain molecules, innate lineage fate of each stem cell population, and the composition of the stem cell niche microenvironment (e.g., nerve tissues and neighboring immune cells)^11,32–35^, which may have tumor promotive or inhibitory functions. Importantly, stem cell differentiation fate can be altered by various physiological and environmental stress factors that change the tumor microenvironment^7,36^. We have reported that stem cell quiescence can act as a tumor suppressor in skin SCC and melanoma formation^23,28^. While hair follicle and melanocyte stem cells can act as cancer cells of origin, when in a quiescent state, these stem cells resist tumor development^23,28^. These studies experimentally demonstrated the importance of extrinsic influences, since tissue injury and/or aberrant stem cell activation can significantly shorten the tumor latency period related to mutant stem cells^23,28^.

In both keratinocyte carcinomas and melanoma skin cancers, a major risk factor is ultraviolet (UV) exposure^37–39^. Numerous studies have determined the effects of UV radiation on skin cancer development. UV radiation can directly act as a mutagen by causing keratinocyte and melanocyte DNA damage^37–39^. However, UV exposure can also act on cancer development and progression indirectly through UV-induced inflammation, which may enhance aberrant activation of tumor-prone but quiescent stem cells harboring pre-existing oncogenic mutations. Experimentally, we have demonstrated that quiescent follicular melanocyte stem cells are less likely to develop cutaneous melanoma even when they express oncogenic mutations, *Braf*^*V600E*^ together with *Pten* loss of function mutations^28,29^. However, during the period of melanocyte stem cell quiescence, cutaneous UV exposure induces aberrant activation and translocation of melanoma-prone stem cells into the interfollicular epidermis, which in turn causes significant malignant melanocytic tumor formation throughout the epidermis^28,29^. Transcriptomic comparison between quiescent melanocyte stem cells and early melanoma cells suggested significant involvement of inflammatory mediators during melanoma formation from tumor-prone stem cells^28^. It has also been reported that cutaneous SCC formation from hair follicle stem cells requires the activation of stem cells. While the oncogenic expression of *Kras*^*G12D*^ along with *p53* loss of function is sufficient to induce invasive SCC formation from hair follicle stem cells, stem cell quiescence significantly inhibits the initiation of SCC formation^23^. Intriguingly, the study found that suppressed tumor formation was dependent on the phosphoinositide 3-kinase–protein kinase B/Akt (PI3K/Akt) pathway, as *Pten* conditional knockout in tumor-prone, quiescent hair follicle stem cells directly induced SCC formation even during the stem cell quiescent period^23^. This suggests that UV radiation can also enhance the aberrant initiation of hair follicle stem cell-originating SCCs since UV radiation is known to activate the PI3K/Akt pathway^40^ and cutaneous SCC formation from murine hair follicles^41^.

Additionally, UV exposure also significantly induces Cox-2 expression and proinflammatory cytokines in the skin^42^. Previous reports have observed that Cox-2 is frequently overexpressed in both keratinocyte and melanoma skin cancers;^1,43–45^ thus, Cox-2 has long been considered a tumor promoter in skin cancers. It is well known that Cox-2 overexpression can increase tumor growth, decrease apoptosis, and advance progression through mechanisms including immune evasion and increased invasiveness through epithelial-mesenchymal transition (EMT)-like processes^46–48^. Experimentally, a rodent diet containing a selective Cox-2 inhibitor had suppressive effects on BCC formation in *Ptch1*^*+/−*^ mice^49^. Similarly, treatment with an NSAID, naproxen, significantly suppressed UV-induced BCC and SCC formation^50^. The role of Cox-2 in skin tumor formation was also supported by transgenic Cox-2 overexpression^51^. Although Cox-2 overexpression alone had no significant association with spontaneous cutaneous SCC tumor formation in genetically engineered mice, transgenic overexpression of Cox-2 demonstrated a significant increase in sensitivity to DMBA/TPA-induced tumor formation in mouse skin^51^. As expected, a significant association of prostaglandin accumulation was observed in the epidermal layers during Cox-2-mediated accelerated tumor formation^51^. Prostaglandin E_2_ is known to bind and activate its G protein-coupled receptors, prostaglandin E_2_ receptors 1 to 4 (known as EP_1_, EP_2_, EP_3,_ and EP_4_). The tumor-promoting role of Cox-2 can partly work through each EP receptor, and inhibition of the receptor pathways has the potential to prevent cutaneous SCCs^52–55^. For example, Tober et al. reported suppressed UV-induced skin tumor formation by treatment with the specific EP_1_ antagonist ONO-8713^52^. Sung et al. reported a significant reduction in DMBA/TPA-induced tumor formation in EP_2_-knockout but not EP_3_-knockout mice^53^, whereas transgenic overexpression of EP_2_ significantly increased cutaneous tumorigenic potential^54^. Similarly, transgenic overexpression of EP4 also significantly enhanced the SCC tumorigenic potential induced by the chemical carcinogen DMBA/TPA and UV radiation in mice^55^.

Importantly, the role of cell-type-specific Cox-2 expression has been determined in cutaneous SCC development. Earlier studies demonstrated that Cox-2 inhibitors suppress UV-induced murine SCC formation^56,57^. Genetic knockout of Cox-2 (prostaglandin-endoperoxide synthase 2 (*Ptgs2*) knockout) also significantly suppressed epidermal differentiation and DMBA/TPA-induced cutaneous papilloma formation^58,59^. More recently, Cox-2 knockout in *Krt14-Cre*^+^ epidermal basal cells induced a significant reduction in chemical carcinogen-induced tumorigenic potential^59^. Similarly, cell-type-specific Cox-2 knockout in *Krt14-Cre*^+^ epidermal basal cells significantly suppressed UV-mediated tumor formation in mouse skin^60^. Using a Cox-2 conditional knockout model (*Ptgs2*^*flox/flox*^) developed by the Herschman group^61^, we have also recently determined the role of cell-type-specific Cox-2 expression in *Krt15-CrePR*^*+*^ tumor-prone hair follicle stem cells expressing *Kras*^*G12D*^ together with a *p53* loss of function mutation^62^. Cox-2 is known to regulate the Akt-mTOR pathway, which is also required for efficient cutaneous wound healing^63,64^. Additionally, Cox-2 inhibition could have suppressive effects on the development of tumors from mutant hair follicle stem cells, as Akt pathway activation enhances SCC formation^41^. Intriguingly, genetic inhibition of Cox-2 suppressed tumor development and inhibited EMT-like properties during tumorigenesis^62^. Oncogenic *Ras/p53* expression significantly and directly induces invasive, mesenchymal-like SCC formation from Krt15-positive stem cells, which tend to show loss of epithelial markers (e.g., E-cad) but overexpression of mesenchymal markers (e.g., vimentin and N-cad)^21,23^. However, the same stem cell populations with a lack of Cox-2 expression tend to form less aggressive or typical epithelial-type SCCs (in contrast to spindle-type SCCs) that are typically well demarcated from the dermis. Together with previous studies^56–60^, this study^62^ demonstrates that Cox-2 expression acts as a tumor promoter that is both required for efficient tumor formation and involved in determining the subtype of cutaneous SCC tumors from the same cancer cells of origin (summary of cell-type-specific Cox-2 expression in cutaneous SCC in Fig. 1b).

In cutaneous melanoma, we have also reported that dexamethasone treatment suppresses UV-induced melanoma formation from mutant, tumor-prone follicular melanocyte stem cells^28^. Dexamethasone is known to cause transcriptional dysregulation by inhibiting the stability of Cox-2 expression;^65^ thus, the inhibitory effects may also act through Cox-2 expression during stem cell-originating melanoma formation. Similar to cutaneous melanomas arising from follicular melanocyte stem cells, a recent study also reported that Krt19-positive hair follicle stem cells could migrate and contribute to SCC formation throughout the interfollicular epidermis upon oncogenic *Hras*^*G12V*^ expression combined with transforming growth factor beta (TGFβ) conditional loss of function, especially during superficial wound healing^66^. Cox-2 is known to be involved in the re-epithelialization of early phases of wound healing;^67^ hence, it is also be important to determine whether selective Cox-2 inhibition can regulate wound healing-mediated hair follicle stem cell migration and SCC formation.

## Cancer cells of origin and Cox-2 in esophageal cancers

### Anatomical and cellular bases of esophageal cancer development

Esophageal cancer is one of the most common lethal cancers worldwide, with an overall 5-year survival rate of up to 20%^4^. Although localized/early-stage tumors have a higher survival rate, the majority of patients present with advanced-stage/metastatic disease. Esophageal cancer prevention research has identified important behavioral risk factors, including smoking and drinking alcohol, and conditions that increase susceptibility, such as gastroesophageal reflux disease (GERD)^68–70^. Recent studies have also determined the cellular diversity of stem/progenitor cells in the esophagus (and murine foregut tissues, which are similarly squamous mucosa) and their potential contribution to esophageal cancer formation^71–75^.

**Fig. 2:**
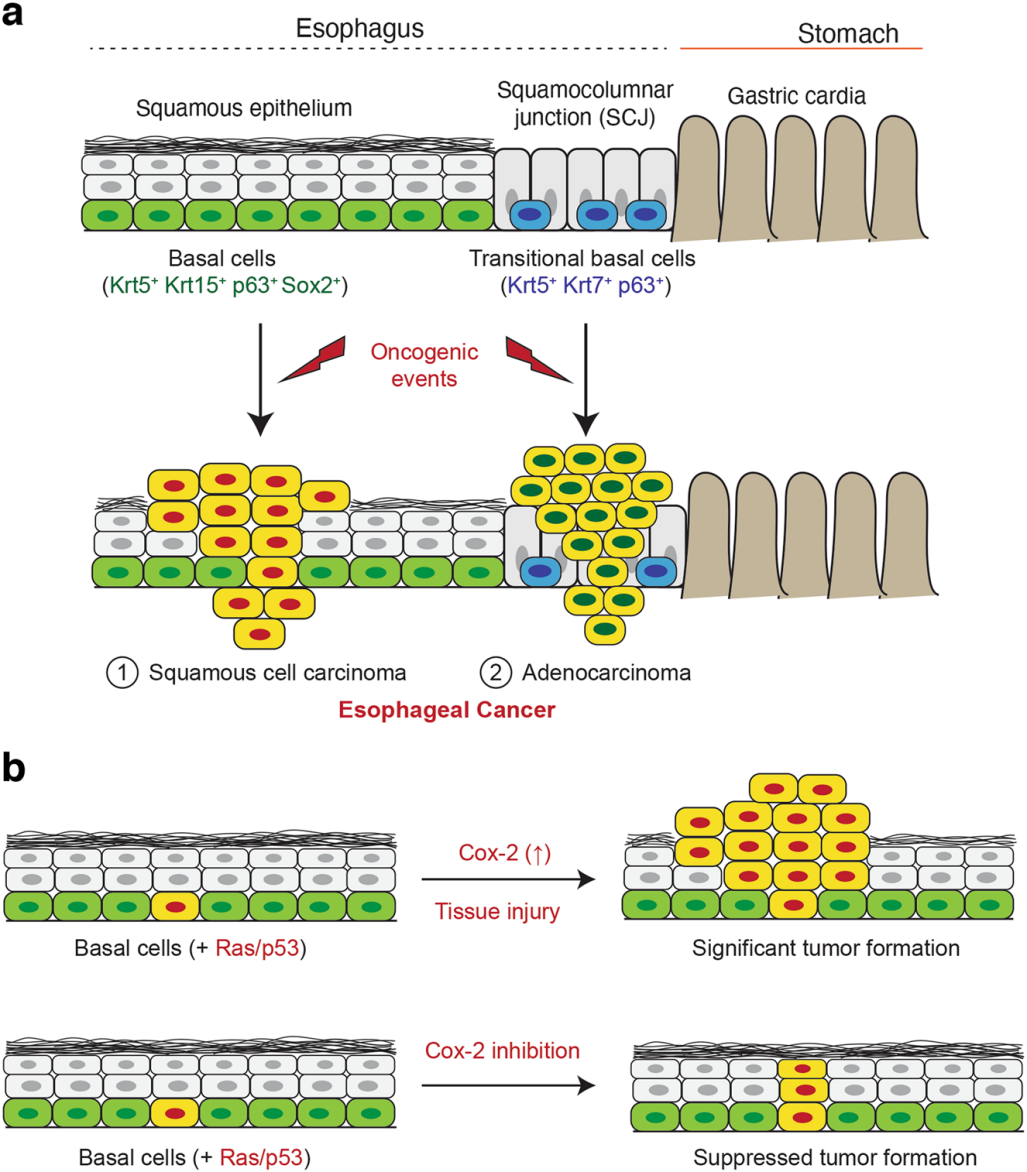
The role of Cox-2 in foregut basal stem/progenitor cells during the earliest stages of SCC formation. **a** Cellular diversity and anatomical distinctions between esophageal SCCs and Barrett’s adenocarcinoma. While SCCs are known to arise from basal stem/progenitors within the squamous epithelium, the cellular origins of Barrett’s adenocarcinoma could be variable. Examples include Krt7-positive transitional basal cells and residual embryonic stem cells at the squamous columnar junctional regions. **b** Cox-2 can be upregulated by cellular extrinsic stress factors such as smoking and gastric acid reflux, which in turn accelerate tumorigenesis. However, Cox-2 inhibition can significantly suppress tumor formation from tumor-prone basal stem/progenitor cells and has features that support a more differentiated cell fate.

There are two distinct types of esophageal cancer: adenocarcinoma, related to mucosal intestinal metaplasia known as Barrett’s metaplasia, and squamous cell carcinoma (SCC). These two cancers are histologically and anatomically distinct. Although still controversial, the cellular origin of adenocarcinomas appears to be distinct stem cell populations located at the squamocolumnar junction^71,72^. In genetically engineered mouse models, histopathological changes such as Barret’s metaplasia can arise from residual embryonic stem cells and/or a Krt7-positive subset of transitional basal stem/progenitor cells^76,77^. On the other hand, SCCs often arise from the squamous layer at the middle to upper (proximal) regions of the esophagus. Oncogenic mutations can induce hyperplastic to papillomatous tumors and SCCs from basal progenitors expressing Krt5, Krt15, p63, and SRY-box2 (Sox-2)^73–75^. In addition to anatomical differences between adenocarcinoma and SCCs (summary diagram in Fig. 2a), the incidence rates of the two tumors also show geographical differences. Adenocarcinoma is increasingly more prevalent in Western countries, including the US^72,78^. Conversely, SCCs are the most common esophageal cancers in Asian countries, and furthermore, esophageal SCCs are one of the main causes of cancer-related deaths in these countries^70,79^. These geographical differences may be due to differences in the level of carcinogenic stress factors such as diet and/or be related to prevalence of smokers and people with GERD within these populations.

### The role of Cox-2 in esophageal tumors

In both esophageal SCCs and adenocarcinomas, the risk factors are related to individual behaviors and other conditions inducing constant tissue injury, which may act as a mutagen and increase tissue turnover rates. In SCCs, smoking cigarettes and drinking alcohol are the most well-known risk factors^70,80^. GERD is a strong risk factor for Barrett’s adenocarcinoma;^68,72,81^ however, GERD is also associated with an increased risk of SCC^74^. These same risk factors are also associated with Cox-2 overexpression;^74,82^ thus, Cox-2 has long been considered a molecular target for esophageal cancer prevention^83–85^.

Experimental studies consistently demonstrate that Cox-2 inhibition has the potential to suppress esophageal SCC formation. In previous studies, the role of Cox-2 in esophageal SCC was tested in an N-nitrosomethylbenzylamine (NMBA)-induced rat SCC model. The oral administration of a selective Cox-2 inhibitor, 4-[4-cyclohexyl-2-methyloxazol-5-yl]-2-fluorobenzenesulfonamide (JTE-522), could inhibit NMBA-induced tumor formation in the rat esophagus^86^. Similarly, a diet containing a selective Cox-2 inhibitor, L-748706 (L-706), suppressed NMBA-induced tumor development^87^. In addition, genetic knockdown of Cox-2 in human esophageal SCC cell lines suppressed tumor formation in vivo in xenograft mice^88^. Importantly, Liu et al. reported that Sox-2-positive basal progenitors contribute to murine foregut tumor formation, and furthermore, Sox-2, inflammation, and signal transducer and activator of transcription 3 (Stat3) cooperate to accelerate malignant transformation of basal progenitors^73^. Stat3 activation in acidic environment-induced inflammation correlates with the activation of Cox-2 and nuclear factor-κB (NF-κB)^89–91^. Cox-2 also facilitates esophageal SCC formation, as Cox-2 expression drives proliferation and reduces apoptosis in epidermal basal layers in skin tissues^58,59^. Notably, our recent study identified a novel role of cell-type-specific expression of Cox-2 in tumor-prone Krt5^+^/Krt15^+^ basal progenitors^74^ using a promoter transgene, *Krt15-CrePR*^92^, and a Cox-2 conditional knockout allele, *Ptgs2*^*flox/flox*61^. Upon expression of the oncogenic Ras/p53 combination (*Kras*^*G12D*^ expression with a lack of *p53* expression), some basal progenitors were able to develop tumors, and tumorigenesis was significantly accelerated by microenvironmental acid-induced stress^74^. However, cell-type-specific knockout of Cox-2 suppresses oncogenic Ras/p53-mediated tumor formation in a genetically engineered mouse model and in 3D organoids^74^. Intriguingly, without significant tumor formation, tumor-prone basal progenitors frequently undergo squamous differentiation and present high expression of the differentiation marker loricrin (primarily expressed in fully differentiated squamous epithelium)^74^. This study demonstrates the importance of inflammation-mediated intrinsic expression of Cox-2 in tumor-prone basal progenitors during esophageal SCC formation. Since Cox-2 can be upregulated in response to various extrinsic and intrinsic stress factors, it is important to note that not only mutations but also inflammatory conditions associated with these stress factors (e.g., inflammation induced by individual behaviors such as smoking cigarettes and drinking alcohol) can further accelerate tumor formation from mutant stem/progenitor cells in a Cox-2-dependent manner. Therefore, controlling Cox-2 expression could be beneficial for preventing esophageal SCC formation in patients who may be prone to constant esophageal tissue injury.

Similarly, constant low pH-induced stress from exposure to gastric and bile acids in GERD patients can induce overexpression of Cox-2 in the esophageal epithelium^70,89^. Cox-2 can also be significantly involved in Barrett’s metaplasia and esophageal adenocarcinoma. While a Cox-2 conditional knockout model has not yet been utilized in genetically engineered murine models of Barrett’s metaplasia (e.g., *Krt7-CreER* models), the role of Cox-2 has been examined in biopsy samples and surgically induced Barrett’s metaplasia rat models. Since bile acid can induce esophagitis in a short timeframe and metaplastic changes in the long term, surgical techniques such as esophagojejunostomy are often used to generate models in both mice and rats. The aim of the surgery is to enhance the exposure of the esophageal mucosa to bile acids. In the surgery-induced model, pharmacological inhibition of Cox-2 has shown potential to inhibit the carcinogenic effects of GERD^85,93,94^. These studies suggest that Cox-2 expression is upregulated in various physiological conditions and involved in pathological changes in both esophageal SCCs and adenocarcinomas.

## Concluding remarks and future directions

Here, we discussed recent studies that explored the cellular origin of cutaneous and esophageal cancers and identified roles for cell-type-specific Cox-2 expression during stem/progenitor cell-originating cancer development. Particularly, these studies demonstrate that Cox-2 can promote the initiation of aggressive tumor formation from tumor-prone stem/progenitor cells in murine skin and enhance the formation of esophageal SCC near the squamocolumnar junction (Figs. 1 and 2). These findings provide new insights into the role of Cox-2 in stem cells and its role in epithelial cancer development. While the majority of studies have focused on Cox-2 in cancer growth and progression, Cox-2 also appears to be important in the initiation of cancers. In addition to in vivo pharmacological inhibition, cell-type-specific Cox-2 suppression and overexpression studies may provide previously unknown information on stem/progenitor cells in the context of tumorigenesis. Additionally, lineage tracing of tumor-susceptible stem cells may further provide data regarding the control of tumor-prone stem cell fate. Genetically engineered mouse models of both cutaneous^62^ and esophageal SCCs^74^ have demonstrated that Cox-2 inhibition promotes a more well- or moderately differentiated phenotype than high levels of Cox-2. A lack or low levels of differentiation are hallmarks of cancers with poorly differentiated phenotypes and poor prognosis. Treatments aimed at stimulating tumor differentiation through the use of agents such as all-trans retinoic acid may be effective in cancer management^95^. It is also possible that all-trans retinoic acid and Cox-2 may have opposite roles or act synergistically in stem cell differentiation, although this has not been documented in cutaneous and esophageal stem/progenitor cells^96^. Therefore, it is important to clearly understand the details of Cox-2 mechanisms in tumorigenesis. Furthermore, whether Cox-2 inhibitors are effective in advanced-stage SCCs by driving differentiation warrants further investigation. Additionally, understanding the role of Cox-2 in cancer stem cells or tumor-propagating cells may also provide useful information for therapeutic targeting. Thus, future studies should define whether Cox-2 can regulate stemness and the differentiation of cancer cells. The effects of targeting Cox-2 may reverse disease status by suppressing stem-like features and enhancing the differentiation status of advanced cancer cells.

We now have a better understanding of how stem/progenitor cells contribute to tumor formation and how inflammation may accelerate tumorigenesis from these tumor-competent stem/progenitor cells. Inflammation is one hallmark of cancer^97^, and Cox-2 is an important mediator of inflammation and cancer. We and others also reported that cell-type-specific Cox-2 knockout in stem/progenitor cells may suppress tumor-promoting inflammation in the niche of cancer cells of origin^60,74^. Suppressed tumor formation by Cox-2 inhibition can be, in part, indirect through a suppressive effect on tumor-promoting inflammation given the crosstalk between immune cells and stem cells. However, the effect of Cox-2 expression on stem cells in their niche needs to be determined in various tumor-initiation sites. While Cox-2 may be a promising molecular target to prevent stem/progenitor cell-originating tumor formation, it is important to clarify whether and how Cox-2 expression in stem cells is required for their normal function and how they regulate the stem cell niche. Importantly, cancer incidence in many organ sites increases with age. This phenomenon may prove to be associated with stem cell aging and Cox-2 expression^98,99^. Therefore, careful measurement of the role of Cox-2 in stem cells and their microenvironmental niche is essential to achieve better preventative methods for skin and esophageal malignancies in aging populations.

Furthermore, understanding the role of downstream molecules of Cox-2 in stem/progenitor cells and stem/progenitor cell-originating cancers may provide better preventative therapeutic targets. While selective Cox-2 inhibitors exist and are FDA approved for some conditions, NSAIDs and Cox-2 inhibitors still have common side effects limiting their long-term use, which is likely required for prevention. Therefore, future studies will need to contribute to a better understanding of Cox-2 pathways, including defining the role of cell-type-specific EP receptors. While this review does not focus on EP receptors in cancers, Cox-2 also acts through EP receptors. Future work should also seek to determine whether selective targeting of EP receptors can provide clinical benefit in stem cell-originating cutaneous and esophageal malignancies in both SCC and non-SCC tumors. Overall, together with identifying the clinical benefit of Cox-2 suppression in skin and esophageal malignancies, future studies should identify what downstream molecules need to be targeted to efficiently maintain normal stem cell functions but significantly inhibit tumor formation from tumor-prone stem cells.
